# Dietary docosahexaenoic acid supplementation prevents the formation of cholesterol oxidation products in arteries from orchidectomized rats

**DOI:** 10.1371/journal.pone.0185805

**Published:** 2017-10-02

**Authors:** Diva M. Villalpando, Mibsam M. Rojas, Hugo S. García, Mercedes Ferrer

**Affiliations:** 1 Departamento de Fisiología, Facultad de Medicina, Universidad Autónoma de Madrid, Madrid, Spain; 2 Unidad de Investigación y Desarrollo de Alimentos, Instituto Tecnológico de Veracruz, Veracruz, México; Max Delbrueck Center for Molecular Medicine, GERMANY

## Abstract

Testosterone deficiency has been correlated with increased cardiovascular diseases, which in turn has been associated with increased oxidative stress. Several studies have considered cholesterol oxidation products (COPs) as oxidative stress biomarkers, since some of them play pro-oxidant and pro-inflammatory roles. We have previously described the cardioprotective effects of a dosahexaenoic acid (DHA) supplemented diet on the aortic and mesenteric artery function of orchidectomized rats. The aim of this study was to investigate whether impaired gonadal function alters the formation of COPs, as well as the potential preventive role of a DHA-supplemented diet on that effect. For this purpose, aortic and mesenteric artery segments obtained from control and orchidectomized rats, fed with a standard or supplemented with DHA, were used. The content of the following COPs: 7α-hydroxycholesterol, 7β-hydroxycholesterol, 7-ketocholesterol, 5,6α-epoxycholesterol, 5,6β-epoxycholesterol, cholestanetriol and 25-hydroxycholesterol, were analyzed by gas chromatography. The results showed that orchidectomy increased the formation of COPs in arteries from orchidectomized rats, which may participate in the orchidectomy-induced structural and functional vascular alterations already reported. The fact that the DHA-supplemented diet prevented the orchidectomy-induced COPs increase confirms the cardiovascular protective actions of DHA, which could be of special relevance in mesenteric arterial bed, since it importantly controls the systemic vascular resistance.

## Introduction

An important number of studies have shown a correlation between increased cardiovascular diseases with testosterone deficiency [1, 2]. It has been demonstrated that sex hormones regulate vascular function since sex hormones deprivation alters the release, function and cell signaling pathways of endothelial factors that could lead to vascular dysfunction. Thus, previous studies from our research group have shown that orchidectomy increased ROS production [3, 4] and prostanoids release [5–7], while the production of nitric oxide [8] and the antioxidant capacity [9–10] were reduced.

The increased oxidative stress has been associated to the development of cardiovascular diseases [11, 12]. Thus, oxidative damage of cellular membranes and enzymes by reactive oxygen and nitrogen species (ROS/RNS) has been described in cardiovascular diseases [13].

Oxidative modification of cholesterol from cell membranes by ROS/RNS leads to the formation of cholesterol oxidation products (COPs), also called oxysterols. COPs can also be formed by enzymatic oxidation and/or by absorption from the diet [14–16]. Regardless of the source, COPs have the ability to induce disruption of fine structure, alteration of integrity, fluidity, and permeability, and loss of biomembrane functionality [17, 18].

Furthermore, oxysterols have been implicated in the pathogenesis of various diseases including cardiovascular diseases, cancer, neurological disorders, and aging [19–22]. COPs are able to modify low-density lipoproteins (LDL) and high-density lipoproteins (HDL) into pro-atherogenic and pro-inflammatory forms. Oxysterols can also trigger pro-oxidative, pro-inflammatory and cytotoxic reactions in the different cell types of the vascular wall, including endothelial cells [23], smooth muscle cells [24–25], fibroblasts [26], monocytes and macrophages [27]. Derived from such evidence, COPs have been proposed as potential biomarkers for non-invasive studies of oxidative stress *in vivo* [28].

Different studies have demonstrated that consumption of Omega-3 polyunsaturated fatty acids (PUFAs) such as eicosapentaenoic acid (EPA) and docosahexaenoic acid (DHA) may reduce the risk of developing cardiovascular diseases [29, 30]. The anti-thrombotic, anti-inflammatory and vasoprotector effects of PUFAs on the cardiovascular system have been reported [31, 32]. Antioxidant propierties of DHA have been also demonstrated since it decreased ROS formation and bound to free radicals preventing tissue oxidation [33–35].

Since it was reported that the lack of sex hormones increases oxidative stress and vascular inflammation, deteriorating factors that could lead to the development of CVD, it is possible that formation of oxysterols in the arterial wall could be modified by the loss of gonadal function. On the other hand, Omega-3 PUFAs may act as a cardioprotective treatment in the oxysterol formation induced by orchidectomy. Therefore, the purpose of the present study was to determine the effects of orchidectomy on the formation of the main oxysterols recognized as products of cholesterol autoxidation [36] (7α-hydroxycholesterol, 7β-hydroxycholesterol, 7-ketocholesterol, 5,6α-epoxycholesterol, 5,6β-epoxycholesterol, cholestanetriol and 25-hydroxycholesterol) in the arterial wall of the aorta and mesenteric artery, as well as the possible preventive effect of a DHA-supplemented diet on COPs formation.

## Materials and methods

### Animals, diets and experimental groups

Male Sprague-Dawley rats (18-week-old) were purchased from Envigo (Mexico City). Rats were housed in stainless steel cages in a temperature-controlled (23 ± 2°C) room, under 12-hour light/dark cycles and standard feeding with fodder and water *ad libitum*. After 4 weeks of adaptation, animals were fed a maintenance diet for rodents (2018S Teklad global 18% protein rodent diets, Envigo, Madison, WI, USA) supplemented with fat (5%). The controls-diet groups were supplemented with sunflower oil (5%) and the DHA groups with 4.7% MEG-3^®^ (Ocean Nutrition Canada Ltd., Dartmouth, NS, CA) and adjusted to 5% with sunflower oil. Nutrient content and energy distribution of each diet is summarized in Table 1. After 2 weeks on the diet, animals were divided into two groups: control and orchidectomized males. Male sex hormone deprivation was induced by orchidectomy at 24 weeks-old under injectable anesthesia with Ketamine-Xylazine (80 mg/kg ket plus 10 mg/kg xil; IP). Rats were treated with 2 mg/kg meloxicam SC (Metacam 5 mg/mL; Boehringer Ingelheim) and with 50 mg/kg ibuprofen immediately after surgery, via IP administration for 5 days. The observation of seminal vesicles atrophy confirmed successful surgery. Animals were maintained under the experimental diets for six more weeks. At the end of the treatment, rats were sacrificed by ether inhalation and decapitation. The mesenteric artery and the aorta were carefully dissected out, cleaned of connective tissue and placed in Krebs-Henseleit solution (KHS) containing, in mmol/L: NaCl 115, CaCl_2_ 2.5, KCl 4.6, KH_2_PO_4_ 1.2, MgSO_4_ 1.2, NaHCO_3_ 25, glucose 11.1, Na_2_ EDTA 0.03 at 4°C. The investigation was performed in compliance with the *Guide for the Care and Use of Laboratory Animals* published by the USA National Institutes of Health (NIH publication No. 85.23 revised 1985), and approved by the Ethical Committee of the *Universidad Autónoma de Madrid* according to directives of the Ministerio de Agricultura, Pesca y Alimentación of Spain (PROEX 202/16).

**Table 1:** Nutrient and energy content of experimental diets.

Macronutrient	Control diet (g/100g)	DHA diet (g/100g)
Crude protein	18.6	18.6
Fat	11.2	11.2
Carbohydrate (available)	44.2	44.2
Crude Fiber	3.5	3.5
Neutral Detergent Fiber	14.7	14.7
Ash	5.3	5.3
Sunflower oil	5.0	0.3
DHA+EPA	0	4.7
Energy Density kcal/100g	310.0	310.0

### Determination of cholesterol and COPs in arterial tissue samples

#### Lipids extraction

Two aortic and two mesenteric rings (*ca*. 3 mm length) from each animal (from the all experimental group of rats) were individually weighed, thawed and subjected to total lipid extraction, as described by Folch *et al*. [37] with some modification [16]. Briefly, arterial rings were homogenized in 1 mL of PBS at 4°C containing BHT (0.05%). Each arterial homogenate was mixed with 16 mL of the Folch solution (chloroform-methanol 2:1 v/v) and 0.01% BHT. Then, 3 mL of NaCl aqueous solution (0.73%) were added. The resulting mixture was separated by centrifugation. The upper phase was discarded and the lipids were collected from the organic layer (lower phase). A mixture of chloroform-methanol-0.73% NaCl (3:48:47 v/v/v, 7.5 mL) was added to the recovered phase and then left at 4°C for 4 h to allow separation. The lower phase was recovered and then filtered through anhydrous sodium sulfate. Subsequently, the extract was resuspended in chloroform with BHT (0.02%) and frozen at -20°C until used.

#### Saponification

Cold saponification was performed according to the method of Menéndez-Carreño *et al*. [38] to remove glycerides, free fatty acids and water-soluble impurities, as well as to release the esterified COPs. For sample preparation, each lipidic extract from the arterial tissue samples was added with 13 μg of the internal standard, 19-hydroxycholesterol (19-OH) (disolved in *n*-hexane:isopropanol, 4:1 v/v) for COPs quantification and with 1 mg of 5α-colestane (disolved in *n*-hexane: isopropanol, 4:1 v/v) as an internal standard for the quantification of cholesterol. The mixture was then dried under stream of nitrogen and mixed with 3 mL of a KOH solution in methanol (4 mol/L or 4 N) with BHT (0.05%), wrapped with aluminium foil and subjected to orbital shaking (300 rpm) at room temperature for 18 h to produce saponification.

For extraction of the unsaponifiable matter, 10 mL of dichloromethane and 5 mL of citric acid (0.1% in double-distilled water) were added to each sample, and mixed vigorously in a vortex. The diethyl ether fraction was then separated by centrifugation. The aqueous layer (supernatant) was discarded and the organic phase was washed with portions of 5 mL citric acid (0.1%, v/v) solution until clear. The organic phase from the samples was dried over anhydrous sodium sulfate. The organic solvent was evaporated with a rotary evaporator at 50°C to remove the dichloromethane. The unsaponifiable extract was dissolved in diethyl ether in a conical vial and dried under nitrogen flow for the subsequent quantification of cholesterol and COPs.

#### Analysis of total cholesterol by gas chromatography

For cholesterol determination, 10% of the unsaponificable matter solution was dried under nitrogen flow and subjected to silylation according to Sweeley *et al*. [39]. A pyridine:hexamethyldisilazane:trimethylchlorosilane (5:2:1, v/v/v) mixture was added (0.3 mL) and heated at 40°C for 20 min. The mixture was then dried under a stream of nitrogen and dissolved in 1 mL of *n*-hexane.

Analyses were performed on an Agilent 7820A gas chromatograph. 1 μL of sample was manually injected in split mode (1:1). Separation of the compounds was performed on a Perkin Elmer PE-5 capillary column (30 m x 0.32 mm d.i. x 1 μm film thickness coated with 5% -phenyl-methylpolysiloxane). The oven temperature was programmed from 280°C to 300°C at a rate of 10°C/min, and mantained for 30 min. The injection and detection port temperatures were set at 325°C. UHP nitrogen was used as the carrier gas at a rate of 1.5 mL/min.

Total cholesterol from the arterial tissue lipidic extracts was quantified by the internal standard method, using 5α-cholestane. Identification of the cholesterol peak in the samples was carried out comparing the retention times with that from the standard. Quantification of cholesterol was performed using a calibration curve.

#### Purification and derivatization of COPs

The remaining 90% of the unsaponificable matter were dried under nitrogen stream and re-suspended in 300 μL of *n*-hexane:ethyl acetate (95:5, v/v) and purified by NH_2_ SPE cartridges, previously equilibrated with 3 mL *n*-hexane deied with anhydrous sodium sulfate at the bottom. The cartridge was eluted with the following solvent sequence: 6 mL of *n*-hexane:ethyl acetate (95:5, v/v), 10 mL of *n*-hexane:ethyl acetate (90:10, v/v) and 10 mL of acetone. The COPs were eluted from the NH_2_ SPE cartridge with the acetone fraction according to the method of Rose-Sallin *et al*. [40]. The purified COPs were dried under nitrogen flow and silylated, according to the method of Sweeley *et al*. [39], and dissolved in 50 μL of *n*-hexane.

#### Analysis of COPs by gas chromatography

One microliter of the silylated COPs was manually injected into the GC under the same conditions used for the determination of total cholesterol. Total COPs were quantified using 19-hydroxycholesterol (19-OH) as internal standard. The COPs identification in the samples was performed by comparison of the retention times with those of the COPs standards and the quantification was performed by calibration curves, as it was done for cholesterol.

### Reagents

All analytical grade solvents and reagents were supplied by Tecsiquim (Mexico City) and from Sigma-Aldrich (Mexico City). For identification and quantification of each COP the internal standards utilized were 19-hydroxycholesterol (internal standard for the quantification of COPs), 5*α*-cholestane (internal quantification standard for cholesterol), Cholest-5-en-3*β*-ol (cholesterol), cholest-5-en-3β-ol-7-one (7-ketocholesterol), 5α,6α-epoxycholestane-3β-ol (5,6α-epoxycholesterol), 5β,6β-epoxycholestane-3β-ol (5,6β-epoxycholesterol), cholestane-3β,5α,6β-triol (cholestanetriol), cholest-5-en-3β,7β-diol (7β-hydroxycholesterol), and cholest-5-en-3β,25-diol (25-hydroxycholesterol). Cholest-5-en-3*α*,7*α*-diol (7*α*-hydroxycholesterol) standard was supplied by Steraloids (Newport, CT, USA).

Solid-phase extraction (SPE-NH_2_) cartridges (500 mg amino-propyl stationary phase/3 mL) were purchased from Phenomenex (Grace Discovery Sciences, Deerfield, IL, USA). The silylation mixture was prepared with dried pyridine, hexamethyldisilazane and trimethylchlorosilane, from Sigma.

### Statistical analysis

All values are expressed as the mean ± SEM (Standard Error of the Mean) of the five animals used. For each value, the mean of the cholesterol and COPs quantification -obtained in each of the two arteries from the same animal- was calculated. Statistical analysis of COPs concentration was performed by one-way analysis of variance (ANOVA) followed by Tukey’s multiple comparison test. For body weight, statistical analysis was done using paired Student’s *t*-test. A *p* value of less than 0.05 was considered significant. Data were analyzed using the GraphPad Software (San diego, CA, USA).

## Results

### Animal body weight

Animals remained healthy and behaved similarly well throughout the experiment. Before the experimental diets (control or DHA) were administered, body weight was evaluated in the four groups of animals, showing no statistical differences among the groups (Table 2). The average weight gain at the end of 8 weeks under the experimental diets was similar for all the experimental groups, which is consistent with our previously published data [9].

**Table 2:** Body weight (g) in control (C) and orchidectomized (ORX) rats fed with a control or DHA-supplemented diet.

	Body weight (g)	
Animal group	Before diet	After diet
C Control	418.5 ± 11	463 ± 10*
C DHA	385.1 ± 12	435 ± 19*
ORX Control	384.6 ± 13	437 ± 12*
ORX DHA	409.2 ± 16	423.4 ± 16*

Values are means ± SEMs. Number of animals per group, n = 5.

*Indicates differences with its respective groups before diets.

### Cholesterol content in aorta

**Fig 1:**
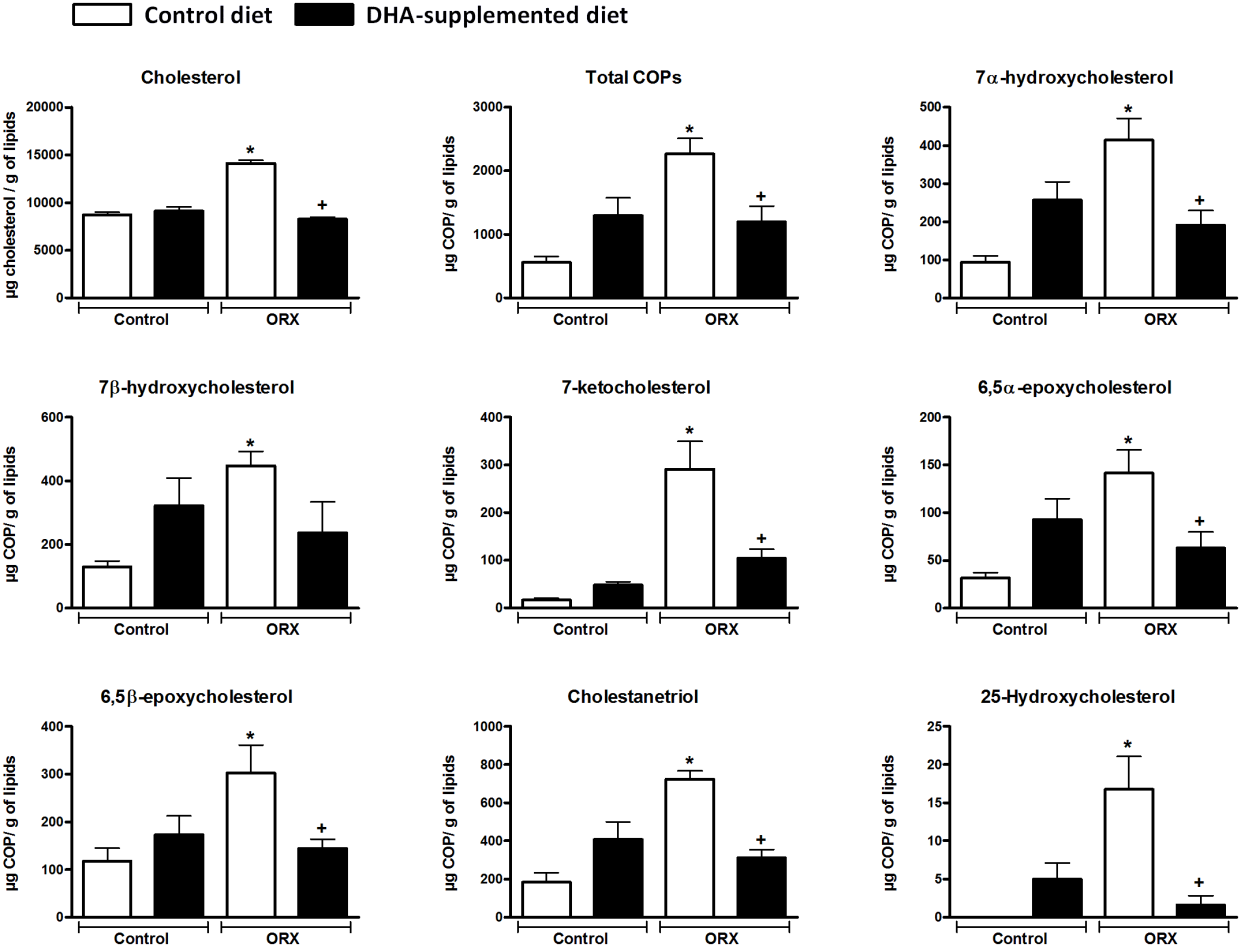
Effect of orchidectomy and DHA suplementation on cholesterol and COPs content in rat aorta. Graphic representation of Chol and COPs (7α-OH, 7β-OH, 7-KC, 5,6β-E, 5,6α-E, 25-OH, CT and total COPs) concentration in μg/g of lipids in aortic rings from control (C) and orchidectomized (ORX) rats fed with a control or with a DHA-supplemented diet. Data were compared using one way ANOVA followed by Tukey`s multiple comparison tests. Values are means ± SEMs. Number of animals, n = 5. *Indicates a *p* < 0.05 *vs* Control group fed control diet. ^+^Indicates a *p*< 0.05 *vs* orchidectomized rats fed control diet.

Orchidectomy significantly increased cholesterol levels in the aortic tissue compared to the control group fed with the control diet (Fig 1). The DHA-supplemented diet caused a significant decrease in cholesterol levels in the aorta from orchidectomized animals. Samples from control rats fed with the DHA-supplemented diet showed similar levels to those found in the control rats fed with the control diet.

### COPs content in aorta

Total COPs levels significantly increased in the orchidectomized group, and decreased in orchidectomized animals fed the DHA-supplemented diet (Fig 1). DHA supplementation did not stastically modify total COPs content in control rats.

Consistent with the above trend, orchidectomy caused a significant increase in the concentration of the studied COPs in aortic segments: 7α-hydroxycholesterol (7α-OH), 7β-hydroxycholesterol (7β-OH), 7-ketocholesterol (7-KC), 5,6β-epoxycholesterol (5,6β-E), 5,6α-epoxycholesterol (5,6α-E), 25- hydroxycholesterol (25-OH), and cholestanetriol (CT) (Fig 1).

The DHA-supplemented diet decreased COPs levels in the aortic tissue of orchidectomized rats, except 7β-OH which did not reach stastistical difference (Fig 1). However, the DHA-diet to control animals tended to increase the concentration of all analyzed COPs.

The proportion in the concentrations of the analyzed COPs in relation to the total COPs was similar in the different groups included in this study. CT was the most abundant COP (*ca*. 30% from total COPs), followed by 7β-OH (*ca*. 20% from total COPs), 7α-OH (*ca*. 18%), 5,6β-E (*ca*. 12%). 7-KC, was higher in both orchidectomized groups (fed with control and DHA-suplemented diet) the ratio remained above the control groups by *ca*. 8–10%. The proportion of 5,6α-E was similar in all groups, being ca. 5% of total COPs. 25-OH could not be detected in the control rats fed the control diet and for the other groups this COP was detected in very small concentrations (≤1%).

### Cholesterol content in mesenteric artery

**Fig 2:**
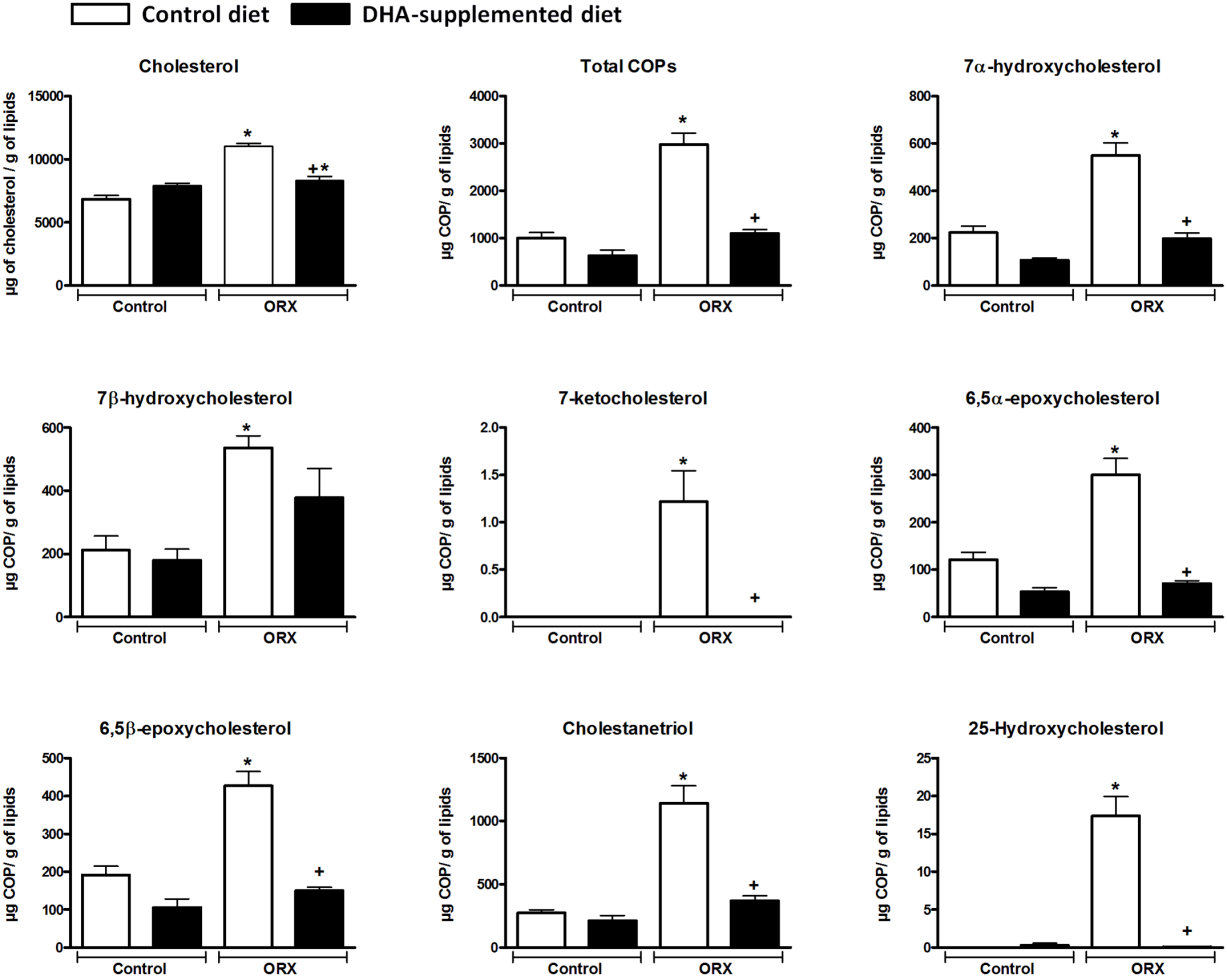
Effect of orchidectomy and DHA suplementation on cholesterol and COPs content in rat mesenteric artery. Graphic representation of Chol and COPs (7α-OH, 7β-OH, 7-KC, 5,6β-E, 5,6α-E, 25-OH, CT and total COPs) concentration in μg/g of lipids in mesenteric artery rings from control (C) and orchidectomized (ORX) rats fed with a control or with a DHA-supplemented diet. Data were compared using one way ANOVA followed by Tukey`s multiple comparison tests. Values are means ± SEMs. Number of animals, n = 5. *Indicates a *p*< 0.05 vs. Control group fed control diet. ^+^Indicates a *p* < 0.05 *vs* orchidectomized rats fed control diet.

The content of cholesterol in mesenteric artery was significantly increased by orchidectomy and the DHA-supplemented diet significantly decreased those levels (Fig 2). The DHA-diet to control rats did not statiscally modify the content of cholesterol in mesenteric artery tissue (Fig 2).

### COPs content in mesenteric artery

Similar results were observed for COPs content in mesenteric artery respect to those found in aorta from these animals. Total COPs content increased significantly in samples from orchidectomized animals, compared to the rest of the groups. The control animals fed with the control diet as well as those fed with the DHA-supplemented diet showed similar COPs levels. Again, DHA supplementation decreased COPs content in orchidectomized animals (Fig 2).

Orchidectomy significantly increased 7α-OH, 7β-OH, 7-keto, 5,6β-E, 5,6α-E, 25-OH, and CT content (Fig 2) compared to the rest of the groups included in the study. As observed in Fig 2, 25-OH content from control rats fed with the control diet was not detected. The same ocurred with 7-KC, which was only detected in minimal amounts in the orchidectomized group, whereas it was practically absent from the mesenteric artery tissue in the other groups.

In the mesenteric arteries from the orchidectomized animals fed with the DHA supplemented diet the concentration of COPs decreased significantly, except for 7β-OH, similarly to that found in aortic tissue (Fig 2). DHA supplementation in control animals tended to decrease the concentration of all the analyzed COPs (7α-OH, 7β-OH, 7-KC, 5,6β-E, 5,6α-E, 25-OH, and CT), unlike the observed in aorta.

Regarding the distribution of COPs content in mesenteric artery tissue, CT was the most abundant COP (*ca*. 30%) followed closely by the 7α-OH and 7β-OH, which showed similar proportions between the same groups, arround 20% of total COPs. Next in proportion is the 5,6β-E representing *ca*. 15% of total COPs, with a slightly higher proportion in the control group (19%). The 5,6α-E represents about 10% of the total COPs, dropping to 6% in the orchidectomized animals fed the DHA-supplemented diet. 25-OH was practically absent, since it could not be detected. In the case of 7-KC, it was only detected in the orchidectomized group at very low concentrations.

## Discussion

This study shows, for the first time, that the loss of gonadal function induced an increase in the formation of COPs in both aorta and mesenteric artery wall. The preventive action of a DHA-supplemented diet on the orchidectomy-induced COPs formation is also demonstrated. These results are in agreement with data previously reported on both the effects of orchidectomy and the beneficial role of a DHA-supplemented diet.

Regarding the results specifically associated to orchidectomy, we have previously reported that the loss of gonadal function of rats induced an overproduction of ROS in aorta [3] and mesenteric artery [4], as well as an increase in the serum content of cholesterol, LDL-cholesterol and triglycerides [9]. It is known that the cholestesterol embeded on the lipid bilayer from the cell membranes is prone to oxidation by ROS [41, 42] which could explain the increased formation of COPs derived from autoxidation processes described in this work. However, oxidation during sample processing can not be ruled out, despite of the utilization of BHT (0.05%). Likewise, it is important to consider that conversion to COPs other than those analyzed may exist. It is well known that the preferential site of oxidation of cholesterol by highly reactive species is at C7 having a relatively weak carbon-hydrogen bond. Moreover, the unique cholesterol double bond between carbons 5 and 6 comprises a vulnerable site for oxidation by free radicals. The most abundant non-enzymatic cholesterol oxidation products present in most tissues are 7α-OH, 7β-OH, 7-KC, and 5,6α-E and 5,6β-E, respectively [43, 44] induced by ROS and RNS [45], which is in line with the findings from the current study, except for 7-KC, which was only detected in mesenteric arteries from orchidectomized rats. CT is one of the most abundant oxysterols, derived from cholesterol by oxidation via formation of 5,6α-E and 5,6β-E as intermediates [46]. The very low detection leves of 25-OH may be attributed to its primarily enzymatic origin, since this oxysterol is generally not considered to be a significant autoxidation product of cholesterol. In addition, the reported levels of 25-OH in most tissues are extremely low [36]. Determination of COPs content can vary depending on the extraction and/or measurement methods, although very low COPs levels under physiological conditions have been reported [47]. In this sense, the 7-KC, 7α-OH and 5,6α-E content -referred to total cholesterol- reported in the present study were similar to those described in human aorta [48] and coronary arteries [49]. However, the relative7-KC, 7α-OH, 7β-OH, 5,6α-E and 5,6 β-E content found in our study are higher than those described in human aorta and carotid artery [49, 50].

On the other hand, COPs are known to trigger oxidative stress by increasing the generation of superoxide anion [51, 52], and down-regulating the expression/activation of Nrf2 [53] Consistent with these results, many studies have reported increased COPs levels in the membranes of cells subjected to oxidative stress, as it has been detected in patients with diabetes mellitus [54], hiperlipidemia [28], chronic inflammatory processes and chronic renal failure [55]. In this regard, the oxysterol clearance mechanisms are probably less efficient in those situations [44], as it was observed in orchidectomized rats in wich the antioxidant activity was decreased [9]. Zhang *et al*. [56] demonstrated that castration decreased enzimatic antioxidant activity (SOD and glutathione peroxidase) and increased malondialdehyde levels, an indicator of lipid peroxidation. In contrast, other studies found that castration did not increase lipid peroxidation [57]. These discrepancies could be attributed to differences in the animal model used, since the maintenance period of gonadectomy determines the induced alterations [8, 58]. However, it is important to emphasize that the experimental model used in this study is the same as that used in our two previous studies [9, 10] and provides relevant information about the key role of the COPs in the orchidectomy-induced modifications of factors, additional to ROS, which regulate vascular function.

It has been mentioned above that different cardiovascular risk factors, such as hypertriglyceridemia, hypertension, diabetes, obesity and overweight, have been associated to COPs content in human serum [15]. In agreement with these results, COPs induced increased blood pressure, serum triacylgycerols as well as body fat index in Wistar rats [59]. On the other hand, the induction of hypertension to rabbits by coarctation of the aorta showed an enhancement of COPs content in both plasma and aortic tissue [60]. In addition, Ares *et al*. [61] indicated that COPs promoted the stimulation of MAPK in human aortic smooth muscle cells, which could partially explain the activation of the MAPK sinaling pathway reported in hypertension after tyrosin kinase receptor transactivation [62]. In this regard, our group has described the activation of MAPK and AKt pathways in mesenteric arteries of orchidedctomized rats, in which the transactivation of EGFR was involved [8]. In this context, these results would indicate that the increase in the formation of COPs may participate in the orchidectomy-induced structural alterations that, in the long term, can lead to the development of hypertension, as it occurs in aged-orchidectomized rats.

Apart from these effects, COPs alter prostaglandin synthesis and stimulate platelet aggregation, an important process facilitating atherosclerosis and thrombosis [42]. Likewise, different COPs induced the expression of COX-2 [63] and the release of prostaglandin E_2_ [64] and prostaglandin J_2_ [65], considered as pro-inflammatory events. Accordingly, it has been described that orchidectomy also leads to a pro-inflammatory environment by increasing prostanoid production in rat aorta [6, 8, 9] and mesenteric arteries [7, 8, 10] most likely as a consequence of increased oxidative stress produced during the experimental conditions.

Nitric oxide is also a critical molecule in vascular function, and the inhibition of endothelial NO-synthase by COPs has been described [52, 66], which is also in agreement with the decreased NO formation observed in aorta [9] and mesenteric artery [10] from orchidectomized rats. Although the decrease of NO production by COPs seems to be demonstrated, studies addressing the influence of COPs in the vascular function have shown contradictory results, since inhibition [67, 68] or no modification [69, 70] of endothelial-dependent vasodilation by 7-KC and 7α-OH-cholesterol have been reported. In our experimental model, orchidectomy did not alter the acetylcholine-induced response in aorta or mesenteric artery, since factor/mechanisms other than NO are simultaneously working to maintain a proper function [4, 7].

These results can be summarized in that orchidectomy induced an increase of the oxidative stress and anti-inflammatory mediators, and a deterioration of the lipid profile, which is accompanied by an increase in the formation of COPs. Interestingly, cholestanetriol -the most abundant COP in all samples, especially in arteries from orchidectomized rats- has been reported to be one of the most cytotoxic oxysterols in different cell lines such as rabbit aortic smooth muscle cells [71], mouse L cells [72], Chinese hamster V79 lung fibroblasts [26], human monocytic cells (U937), colonic adenocarcinoma cells (CaCo-2) and hepatoma liver cells (HepG2) [73]. However, it is important to point out the cytotoxic effects reported for different COPs other than cholestanetriol, also in different cell lines, related to vascular and nervous systems that contribute to the pathogenesis of cardiovascular [52, 74] and neurodegenerative [19, 20] diseases.

It has been suggested that generation of some oxysterols can be reduced in the presence of antioxidants [43] by scavenging ROS. For instance, supplementation of vitamin E to diabetic patients can decrease 7-KC and 7β-OH levels [75]. Likewise, Uemura *et al*. [76] found that apoptosis induced by 7β-OH and 7-KC in vascular endothelial cells was prevented by α-tocopherol. DHA exhibits potent anti-oxidant properties which attenuate ROS overproduction in endotelial cells [77] and enhances the overall antioxidant status [78]. Recently, the ability of DHA to prevent ROS overproduction and oxiapoptophagy induced by 7KC, 7β-OH, and 24-OH in oligodendrocytes was demonstrated [79]. De Medina *et al*. [80] found that DHA inhibited cholesterol-5,6-epoxide hydrolase activity, that catalyzes the conversion of 5,6α-E and 5,6β-E to its product CT, which exerts important citotoxic effects. Also, DHA showed protective effects on COPs-induced cell death [33]. These results agree with our findings, since the DHA-supplemented diet to orchidectomized rats prevented the rise in COPs formation caused by orchidemtomy. Thus, the levels of COPs were near to those measured in control rats fed the control diet. Interestengly, the DHA-supplemented diet restored the increased contents of COPs induced by orchidectomy, similarly how it restores the lipid profile, and the redox and inflammatory status altered in this particular condition [9,10]. These results are in agreement with the cardioprotective effects of DHA widely reported through its anti-inflammatory [31] and anti-oxidant activities [33–35]. It has been recently reported that DHA, one of the main fatty acids of the Mediterranean diet, attenuates the 7-KC-induced toxic effect on microgial cells [81]. Based on all this information, human dietary habits deserve important consideration and comsumption of a Mediterranean diet could prevent the consequences of an increased oxidative stress that occurs in diverse physiopathological situations. However, something different seems to occur with the DHA-supplemented diet to the control animals, in which a tendency to increase COPs content was observed in the rat aorta. This result is in line with a previous report, in which the involvement of vasodilator prostanoids in the Ach-induced response in aorta from control rats was switched to vasoconstrictors after the DHA-suppelemented diet [9]. Overall, these results indicate that DHA-supplementation exerts cardiopotective effects, especially in pathological conditions, which seems to be reasonable since the homeostatic mechanisms work to maintain proper function in healthy subjects without any nutritional supplement.

In summary, we describe here the detrimental effects caused by the lack of sex hormones in the rise in lipid peroxidation, providing new information on the damage on lipid components responsible of maintaining membrane properties and cell signaling in different pathways involved in the vascular function of aorta and mesenteric artery, already published [9,10]. The preventive effect of a DHA-supplemented diet on lipid peroxidation, which promotes the maintenance of vascular homestasis, is also shown. In this regard, it is important to note that the results observed in the mesenteric artery, together with those related to vasomotor function [10], are of special relevance since this vascular bed importantly contributes to the control of the peripheral vascular resistance and therefore to the regulation of blood pressure. The present findings reveal the interest in conducting clinical studies with DHA in patients with decreased levels of sex hormones (elderly patients and/or prostate cancer patients undergoing androgen deprivation therapy) who display cardiovascular risk factors.
